# Freeze Drying of Polymer Nanoparticles and Liposomes Exploiting Different Saccharide-Based Approaches

**DOI:** 10.3390/ma16031212

**Published:** 2023-01-31

**Authors:** Ilaria Andreana, Valeria Bincoletto, Maela Manzoli, Francesca Rodà, Vita Giarraputo, Paola Milla, Silvia Arpicco, Barbara Stella

**Affiliations:** Dipartimento di Scienza e Tecnologia del Farmaco, Università di Torino, Via P. Giuria 9, 10125 Torino, Italy

**Keywords:** nanoparticles, liposomes, freeze drying, cryoprotectants, hyaluronic acid

## Abstract

Biodegradable nanocarriers represent promising tools for controlled drug delivery. However, one major drawback related to their use is the long-term stability, which is largely influenced by the presence of water in the formulations, so to solve this problem, freeze-drying with cryoprotectants has been proposed. In the present study, the influence of the freeze-drying procedure on the storage stability of poly(lactide-*co*-glycolide) (PLGA) nanoparticles and liposomes was evaluated. In particular, conventional cryoprotectants were added to PLGA nanoparticle and liposome formulations in various conditions. Additionally, hyaluronic acid (HA), known for its ability to target the CD44 receptor, was assessed as a cryoprotective excipient: it was added to the nanocarriers as either a free molecule or conjugated to a phospholipid to increase the interaction with the polymer or lipid matrix while exposing HA on the nanocarrier surface. The formulations were resuspended and characterized for size, polydispersity index, zeta potential and morphology. It was demonstrated that only the highest percentages of cryoprotectants allowed the resuspension of stable nanocarriers. Moreover, unlike free HA, HA-phospholipid conjugates were able to maintain the particle mean size after the reconstitution of lyophilized nanoparticles and liposomes. This study paves the way for the use of HA-phospholipids to achieve, at the same time, nanocarrier cryoprotection and active targeting.

## 1. Introduction

Applications of nanoparticulate systems for drug delivery have been extensively discussed over the years for their promising therapeutic and diagnostic purposes [1,2,3]. Indeed, nanocarriers make it possible to overcome the bioavailability hurdles of poorly water-soluble drugs, improve pharmaceutical efficacy and prevent significant side effects by modulating their physico-chemical characteristics [4,5]. However, formulation and long-term storage of drug delivery systems can be challenging [6]. Lipids or polymers used for developing biodegradable nanocarriers in aqueous solutions are usually affected by chemical and/or physical instability. Hydrolysis of nanoparticles, which may lead to polymer degradation and loss of encapsulated drug, and aggregation frequently occur when the nanosystems are stored as aqueous suspensions for an extended period [7]. To control the nanoscale morphology and avoid alteration of the final suspension, surfactant agents can be added to prevent particle agglomeration, although the potential toxicity can limit their application. As an alternative, the coating of the particle surface with a surrounding shell has been proposed: the stability of a liposome-based formulation was enhanced by a coating of chitosan that protected liposomes from rapid degradation and improved controlled drug release [8]. As an alternative, to increase particle stability, the nanocarriers can be formulated as dry powders to restrict Brownian motion and reduce the chance of particles aggregating upon storage.

**Figure 1:**
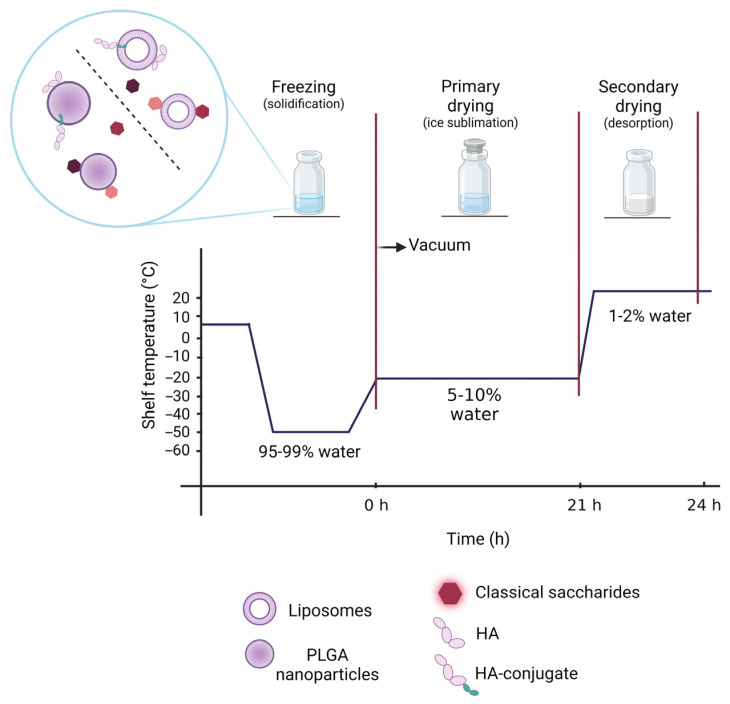
Schematic representation of the freeze-drying process for PLGA nanoparticles and liposomes formulated with different cryoprotectants (created with BioRender.com).

Freeze drying, also named lyophilization, is the most used method to remove water and handle the long-lasting stability of nanoparticle formulations [9,10]. As an example, over the last two years of the COVID-19 pandemic, the urgent need to achieve a dry form of nanoparticle-based therapeutics highlighted the importance of lyophilization studies to improve the storage stability of lipid nanoparticle vaccines [11,12]. Freeze drying removes water from a frozen sample by sublimation and desorption under a vacuum in three main steps: freezing, primary drying and secondary drying (Figure 1). The first freezing phase is the key step known to generate a variety of stresses, which have a significant impact on nanoparticle stability concerning aggregation and formation of macroscopic particles [13,14,15]. Furthermore, the freeze-drying procedure may alter drug content, particle size distribution and production of a workable cake with a short reconstitution time [16,17]. For these reasons, to protect the formulations from possible alterations induced by the dehydration process, it is advisable to add some excipients that act as protectants during freeze drying [18]. The excipients routinely employed include cryo- and/or lyoprotectants that are chemically innocuous and protect the nanoparticles during the freezing or drying stage of the process, respectively. The addition of protective excipients should depend on the nature of nanoparticles and the different components added during the formulation. For instance, as cited before, polymer-based nanoparticles can be formulated with the addition of surfactant agents, such as polyvinyl alcohol (PVA), that can protect the system during the freeze-drying process [19,20]. However, free-surfactant formulations require cryo- or lyoprotectants to retain nanoparticle physico-chemical properties.

Since they show the ability to act as integrity membrane protectants, short-chain saccharides are the preferable aid-molecules useful to minimize nanoparticle instability during the freezing steps of lipid and polymer nanoparticles [6,21,22]. They act by replacing the bound water around nanocarriers and forming a viscous matrix which reduces their mobility through the process [23,24]. Different theories have been proposed to explain the stabilization effects of saccharides during lyophilization [25,26]. Thanks to their influence on the polymer glass transition temperature, they are the most commonly used excipients to obtain an amorphous form, a suitable redispersibility and good drug stabilization of dried samples [27]. Classical saccharides, such as sucrose, glucose and mannitol, proved to stabilize various nanosystems by protecting them during the freezing step and/or during the drying step and/or storage [28]. It was demonstrated that mono- and disaccharides provided resuspended nanoparticle stability thanks to their flexibility, which let them be less affected by steric hindrance. However, oligo-/polysaccharides and short-chain sugars may have a synergistic action in cryoprotection and water replacement [29].

Besides the reported studies on conventional cryo- and lyoprotectants, to the best of our knowledge, a comparative study between polymer nanoparticles and liposomes stabilized with short and long-chain saccharides has not been assessed yet. To this aim, we selected sucrose, trehalose, mannitol and glucose as cryoprotectants and tested their protective activity on poly(lactide-*co*-glycolide) (PLGA) nanoparticles and liposomes at different concentrations. Moreover, a comparative study with hyaluronic acid (HA) at a low molecular weight (4.8 kDa and 14.8 kDa) as a free saccharide or conjugated to a phospholipid [30] was assessed to investigate the influence of HA interactions with liposome or PLGA nanoparticle matrix in obtaining a workable dried product in which HA is stably associated to the nanocarriers (Figure 1).

## 2. Materials and Methods

### 2.1. Materials

All the phospholipids, cholesterol (CHOL), PLGA 50:50 (Resomer^®^ RG 502 H, 7–17 kDa), PLGA 75:25 (Resomer^®^ RG 752 H, 4–15 kDa), PEG_2000_-PLGA 50:50 (PLGA 11.5 kDa), D-(+)-glucose, D-(+)-sucrose, D-(+)-trehalose, D-(+)-mannitol (purity minimum 95%) and solvents (analytical grade) were purchased from Merck (Milan, Italy). Sodium hyaluronate (HA) (4.8 kDa or 14.8 kDa) was purchased from Lifecore Biomedical (Chaska, MN, USA). The compound 1,2-dipalmitoyl-*sn*-glycero-3-phosphoethanolamine (DPPE) was conjugated to 4.8 kDa or 14.8 kDa HA (HA_4.8_-DPPE or HA_14.8_-DPPE) using the method described by Arpicco et al. [30]. Filtered MilliQ^®^ water (Millipore, Merck) was used. Solvents were evaporated using a rotating evaporator (Heidolph Laborota 400, Heidolph Instruments, Schwabach, Germany) equipped with a vacuum pump (Diaphragm Vacuum Pump DC-4).

### 2.2. Preparation of Nanoparticles and Liposomes

PLGA nanoparticles were prepared by the nanoprecipitation technique [31]. To this aim, 6 mg of PLGA (50:50 or 75:25) were dissolved in 500 µL of acetone. The organic solution was then poured into 1 mL of MilliQ^®^ water under magnetic stirring. Precipitation of nanoparticles occurred spontaneously without using any surfactant. After solvent elimination under rotary evaporation, an aqueous suspension was obtained (PLGA concentration: 6 mg/mL). Further batches were prepared by 2:1 (*w*/*w*) polymer blends between PLGA (50:50 or 75:25) and PEG_2000_-PLGA 50:50, as previously described (total polymer concentration: 6 mg/mL) [32].

Liposomes were prepared using the thin lipid film-hydration method by mixing 1,2-dipalmitoyl-*sn*-glycero-3-phosphocholine (DPPC), CHOL and L-α-phosphatidylglycerol (PG) in 70:30:3 molar ratio. Lipids were dissolved in chloroform and then evaporated by a rotary evaporator. The resulting thin film was dried under vacuum overnight and then hydrated with 900 µL of 4-(2-hydroxyethyl)piperazine-1-ethane-sulfonic acid (HEPES) buffer 0.02 M pH 7.4, vortexed and bath sonicated. Afterward, the obtained suspension was extruded (Extruder, Lipex, Vancouver, BC, Canada) at 60 °C under nitrogen through a 200 nm-polycarbonate membrane (Costar, Corning Inc., New York, NY, USA).

All the suspensions were stored at 4 °C until further use.

### 2.3. Preparation of HA-Decorated Nanoparticles and Liposomes

For PLGA nanoparticles, HA_4.8_-DPPE or HA_14.8_-DPPE conjugates were added in the aqueous phase before the addition of the polymer acetone solution during the nanoprecipitation procedure. Practically, 3.1 mg of HA_4.8_-DPPE or 15.3 mg of HA_14.8_-DPPE were added to 1 mL of MilliQ^®^ water. Then, for each preparation, 6 mg of PLGA (50:50 or 75:25) were dissolved in 500 µL of acetone and poured into the aqueous phase containing the HA derivative under magnetic stirring. Acetone was then eliminated under rotary evaporation.

HA-decorated liposomes were prepared as described above for plain liposomes by adding to the phospholipid mixture (composed of DPPC/CHOL 70:30 molar ratio) HA_4.8_-DPPE or HA_14.8_-DPPE conjugates (3 molar ratio) in 900 μL of HEPES buffer during the hydration of the lipid film.

### 2.4. Freeze-Drying Studies

Once the development of nanosystems was optimized, PLGA nanoparticles and liposomes (both blank and HA-decorated) were freeze-dried with an Alpha 1–4 LSCplus freeze-drier (Martin Christ, Osterode am Harz, Germany). In addition, different saccharides (mannitol, glucose, sucrose, trehalose, free HA) were evaluated as cryoprotective agents for blank nanoparticles or liposomes.

In particular, for PLGA nanoparticles, mannitol, glucose, sucrose or trehalose were added at the concentration of 2.5, 5, 10 or 20% (*w*/*w*) either in the aqueous phase prior to the addition of acetone to the aqueous solution or after the evaporation of the organic solvent. Moreover, the addition of free HA was compared with the insertion of HA-DPPE conjugates: to this aim, free 4.8 kDa HA (1.31 mg) or free 14.8 kDa HA (3.95 mg) were added to nanoparticle suspensions after organic solvent evaporation.

Sucrose, trehalose and free HA were selected to test the cryoprotective effect on liposomes. Sucrose and trehalose were added directly both to the hydration buffer and after liposome formation in a 5:1 (*w*/*w*) sugar:lipids ratio or only to the resulting liposome suspensions. Free HA was added in the same conditions in a 3% molar ratio.

For all the formulations, the freeze-drying program consisted of an initial freezing of the samples in liquid nitrogen. After that, the freeze-dryer was pre-cooled at −54 °C and samples were introduced therein. Then, the temperature of the shelf was increased to −20 °C for 21 h with a chamber pressure of 0.1 mbar to remove ice by sublimation (primary drying). Finally, a secondary drying step was carried out at 25 °C and 0.010 mbar for 3 h.

To test the cryoprotective effect, freeze-dried formulations were then resuspended by vortex-mixing (and bath sonication for PLGA nanoparticles) in the same volume of MilliQ^®^ water or HEPES buffer as before freeze-drying.

### 2.5. Physicochemical Characterization of Nanoparticles and Liposomes

The mean particle hydrodynamic diameter and the polydispersity index (PDI) of the different nanoparticle and liposome samples were determined at 25 °C by quasi-elastic light scattering (QELS) using a nanosizer (Nanosizer Nano Z, Malvern Inst., Malvern, UK). The selected angle was 173°, and the measurement was made after 1/10 dilution of the particulate suspensions in MilliQ^®^ water. Each measurement was performed in triplicate both before and after the freeze-drying process. The particle surface charge of the formulations was investigated by zeta potential measurements at 25 °C using the Smoluchowski equation and the Nanosizer Nano Z after 1/10 dilution of the suspensions in MilliQ^®^ water. Each value reported is the average of three measurements both before and after the freeze-drying process.

Finally, the physical stability of resuspended nanoparticles and liposomes was determined in the storage conditions (4 °C) by evaluating mean hydrodynamic diameter, PDI and zeta potential at different interval times during 4 weeks at 4 °C (measures performed after 7, 14, 21 and 28 days).

In order to have information on the morphology and size of the HA-decorated nanoparticles and liposomes, Field Emission Scanning Electron Microscopy (FESEM) analyses were performed by a Tescan S9000G FESEM 3010 microscope (Tescan Orsay Holding a. s., Brno-Kohoutovice, Czech Republic) working at 30 kV, equipped with a high brightness Schottky emitter and fitted with Energy Dispersive X-ray Spectroscopy (EDS) analysis by an Ultim Max Silicon Drift Detector (SDD, Oxford, UK). HA_4.8_-DPPE-decorated nanoparticles and liposomes were examined both before and after the freeze-drying process as well as after further resuspension. For analyses, one drop of the as prepared and resuspended samples was placed on an aluminum stub coated with a conducting adhesive and left to dry in air at room temperature. As for the freeze-dried samples, the powder was briefly contacted with the same kind of stub described previously. All samples were then simply submitted to metallization with Cr (ca. 5 nm) to avoid any charging effect (Emitech K575X sputter coater, Quorumtech, Laughton, East Sussex, UK) and inserted in the chamber by a fully motorized procedure.

## 3. Results and Discussion

**Figure 2:**
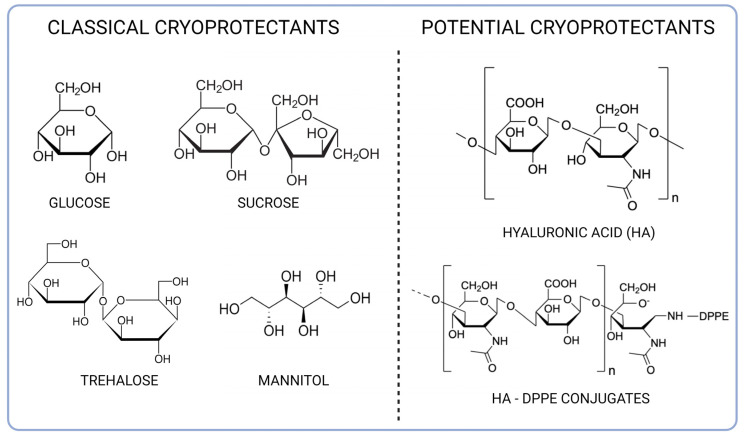
Chemical structures of the excipients used in this study.

Freeze drying allows drug delivery systems, such as nanoparticles and liposomes, to be obtained and stored as dry forms, thus avoiding chemical and physical instability and improving long-term storage [33,34]. However, several factors can lead to colloidal instability during the freeze-drying process, and many strategies are considered to avoid particle aggregation [7]. Among them, the use of cryoprotectants to embed nanocarriers in an amorphous matrix is one of the most used approaches, and saccharides are largely used to prevent aggregation and facilitate redispersion of the lyophilized powder [6]. Nevertheless, the choice of the saccharide and the conditions of use are crucial for the process, and they differ according to the considered nanosystem. On these bases, the objective of the present study was the preparation of PLGA nanoparticles and liposomes in the presence of various saccharides (monosaccharides, such as mannitol and glucose, disaccharides, such as sucrose and trehalose, and a polysaccharide, HA) to evaluate the cryoprotective effect on the selected nanosystems (Figure 2). Furthermore, concerning HA, it was added to nanoparticles and liposomes in its free form and also as DPPE-conjugates to allow a stronger association with both nanoparticles and liposomes.

**Figure 3:**
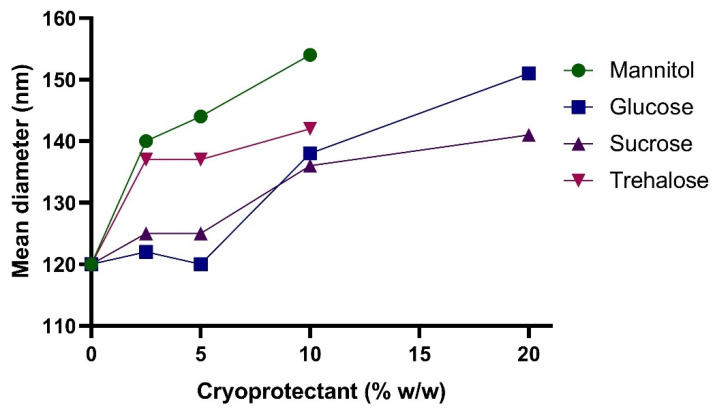
PLGA 75:25 nanoparticle mean hydrodynamic diameter as a function of the percentage of the different cryoprotectants when added before pouring the acetone solution into the aqueous phase (*n* = 3, S.D. < 10% for all samples).

Glucose, sucrose, trehalose and mannitol were added between 2.5% and 20% (*w*/*w*) to PLGA or PEG_2000_-PLGA nanoparticle samples according to the percentages reported in the literature [18]. In particular, in the first set of experiments with PLGA 75:25, sugars were added either to the aqueous phase before the nanoprecipitation process or immediately after the elimination of organic solvents by rotary evaporation, based on previous findings [35]. Then, the nanoparticles were characterized before freeze-drying in terms of mean hydrodynamic diameter and PDI. Results showed that, although the PDI value was less than 0.1 for all the samples, the mean hydrodynamic diameter trend was different according to the preparation procedure. Indeed, when the cryoprotectants were added to the aqueous phase before pouring the organic solution, the mean hydrodynamic diameter tended to increase as a function of the saccharide concentration, as shown in Figure 3. The presence of sugars before the spontaneous formation of nanoparticles can probably lead to sugar encapsulation into the polymer matrix, thus increasing the mean size and probably competing with a potential active principle [36]. On the contrary, when the cryoprotectants were added after the nanoparticle formation (i.e., after solvent evaporation), the mean hydrodynamic diameter did not vary as a function of the sugar concentration, although a 10–20% increase for glucose, trehalose and mannitol was observed. Thus, considering the size analysis, we selected the sugar addition after nanoparticle formation as the most efficient preparation method for PLGA nanoparticles. Indeed, even with PLGA 50:50, the mean hydrodynamic diameter did not change in these conditions.

Again, for PEGylated nanoparticles prepared from a blend of PLGA (75:25 or 50:50) and PEG_2000_-PLGA, the cryoprotectants were added to the formulations after solvent evaporation. Even in this case, the mean hydrodynamic diameter was not affected by the addition of the saccharides, although the particle size was smaller than that of pure PLGA nanoparticles and comprised in the range 85–105 nm, probably due to the amphiphilic nature of PEG_2000_-PLGA.

The zeta potential values measured for all PLGA nanoparticles were found in the range between −36 mV and −21 mV, regardless of the nanoparticle composition; these values allow nanoparticles to be stable thanks to charge repulsion.

PLGA nanoparticles were then freeze-dried without cryoprotectants or in the presence of glucose, sucrose, trehalose or mannitol at different concentrations. Particle characterization was then carried out on samples resuspended in MilliQ^®^ water. All the samples could be resuspended except for nanoparticles without cryoprotectant and those with mannitol, which gave a precipitate. After lyophilization, these samples appeared as a very compact powder, which probably hampered the rehydration. On the contrary, the formulations with glucose, sucrose or trehalose were able to be resuspended. Nevertheless, only the highest percentages of sucrose and trehalose (reported in Table 1) allowed nanoparticles to retain the size they had before freeze-drying. As reported elsewhere, the level of stabilization afforded by saccharides often depends on their concentration [37]. Indeed, the lowest percentages of cryoprotectants did not avoid particle aggregation after resuspension [24]. However, high concentrations of sugars (e.g., up to 25% of glucose and trehalose) could not stabilize nanoparticles, and they may even destabilize them [38]. Furthermore, all glucose-associated PLGA 75:25 nanoparticles showed a mean hydrodynamic diameter at least three times higher than that before freeze-drying (an exception was represented by PLGA 50:50 nanoparticles freeze-dried in the presence of glucose, which retained the size in the presence of 10% glucose). With PEG_2000_-PLGA in the polymer matrix, even at a high percentage of cryoprotectants, the mean hydrodynamic diameter and PDI increased, probably due to the interaction of sugars with the outer PEG layer [16,39]. Finally, the samples with the highest percentages of cryoprotectants were also stable for 4 weeks after resuspension during storage at 4 °C.

Concerning zeta potential measurements after resuspension, for polymer nanoparticles, the overall values were found to be slightly more negative than those of the suspensions before freeze drying (between −41 mV and −27 mV).

The effect of disaccharides as protective agents in the freeze-drying process was also assessed on liposomes. Liposomal formulations were prepared by hydration of the lipid film followed by extrusion through polycarbonate filters to obtain homogenous small unilamellar vesicles. Sucrose and trehalose were investigated for their ability to prevent aggregation of liposomes after freeze-drying [23] since disaccharides are considered the excipients of choice to stabilize the liposome membrane [26]. Similar to the nanoparticle-based approach, cryoprotectants were added to the hydration buffer and after liposome formation (“in/out” addition method) or directly in the final liposomal suspension (“out” addition method). As shown in Table 2, the two additional methods demonstrated no important variations in liposome size, and their surface charge remained slightly similar (around −15 mV). For liposomes, the sugar:lipid ratio of 5:1 (*w*/*w*) was used since it was reported to be able to maximize the stabilization effect, in particular in the case of disaccharides [40]. On the contrary, in the case of monosaccharides, a higher ratio (9:1) is generally required to preserve the physical integrity of resuspended liposomes [41].

Thus, to set a comparative study between polymer and lipid nanoparticles, further tests were performed by adding the cryoprotectants after nanosystem formation (“out” addition method). As a first result, we confirmed that, as for polymer nanoparticles, for lipid-based nanocarriers, significant amounts of cryoprotectants are required to supersaturate the surface and lead to the formation of a glassy matrix all around their structure for efficient carrier protection during lyophilization [42].

**Figure 4:**
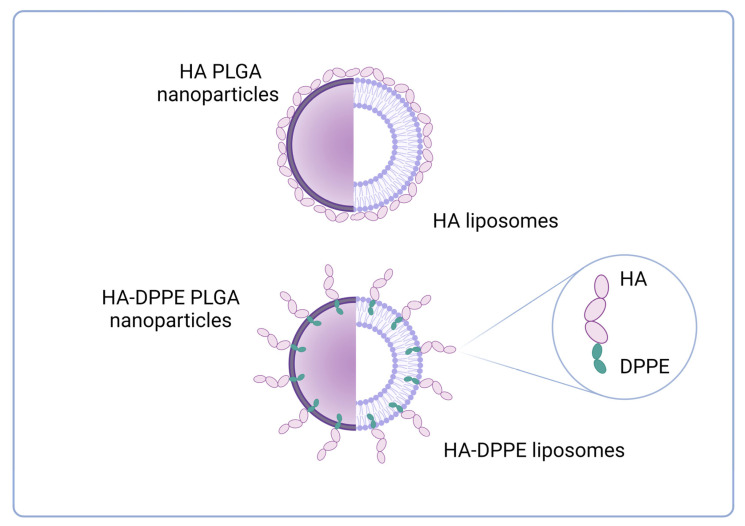
Schematic representation of the association of HA and HA-DPPE conjugates to polymer and lipid nanocarriers (created with BioRender.com).

After considering different sugars, we moved towards the use of a polysaccharide, namely HA, which is an anionic glycosaminoglycan already studied for the cryopreservation of cell membranes [43]. Furthermore, HA has received particular attention for its interesting biological and/or pharmacological properties and as a potential active targeting agent for the CD44 receptor [44,45,46]. In this work, free HA was added to the nanocarrier suspensions to assess its protective activity during the freeze-drying process. Furthermore, HA of different molecular weights was also conjugated to DPPE to allow the insertion of the lipophilic DPPE chains into the polymer or lipid matrix, thus achieving a more stable association of HA to the nanocarriers (Figure 4).

Free HA (4.8 kDa or 14.8 kDa) was directly added to the final PLGA (75:25 or 50:50) nanosuspensions, and then the freeze-drying procedure was carried out. After the addition of water, lyophilized samples could not be resuspended, as they formed large aggregates. We can thus highlight that free HA at the two considered molecular weights does not have a protective effect on PLGA nanoparticles during the freeze-drying process.

**Figure 5:**
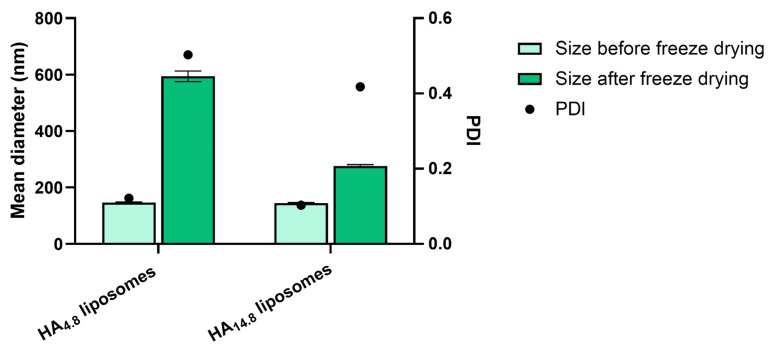
Particle hydrodynamic diameter and PDI of liposomes before and after freeze-drying in the presence of free HA (*n* = 3).

The potential cryoprotectant effect of free HA was also assessed on liposomes. As for nanoparticles, HA was added directly to the final liposomal suspensions. Characterization of liposomes after freeze-drying in the presence of free HA showed a weak cryoprotectant effect for HA 14.8 kDa. On the contrary, a significant size increase was observed for HA at a lower molecular weight (Figure 5).

**Figure 6:**
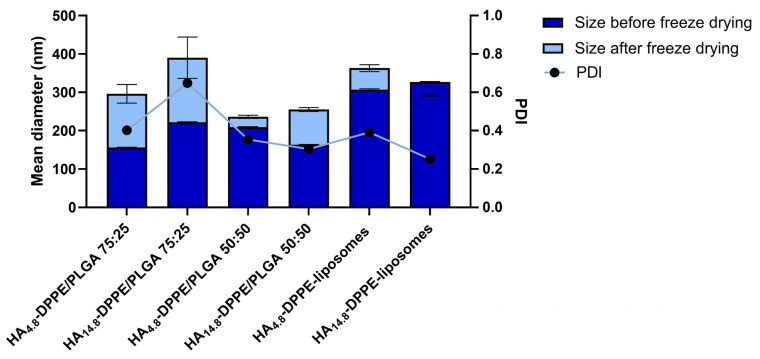
Mean particle hydrodynamic diameter and PDI of rehydrated freeze-dried PLGA nanoparticles and liposomes with HA-DPPE conjugates (*n* = 3).

In the further set of experiments, we evaluated the effect of HA as a cryoprotectant agent when it is associated with polymer- and lipid-nanocarrier structure by hydrophobic interactions. Since it is highly hydrophilic, HA (4.8 kDa or 14.8 kDa) was linked to the DPPE phospholipid, which is able to associate with a polymer matrix or phospholipid bilayer and obtain HA-coated nanoparticles. These conjugates have already been used in several settings to prepare HA-decorated liposomes for cell targeting that have been proposed for either intravenous or cutaneous administration [47,48,49,50]. For PLGA nanoparticles, HA-DPPE conjugates were added into the aqueous phase since they are not soluble in acetone, while for liposomes, conjugates were added during the hydration of the lipid film. The potential activity of HA-DPPE conjugates as cryoprotectants during lyophilization was assessed by analyzing the particle size and PDI of PLGA nanoparticles and liposomes before and after freeze drying (Figure 6). All formulations in the presence of HA-DPPE conjugates could be easily redispersed after freeze-drying. In particular, the mean hydrodynamic diameter of PLGA 50:50 nanoparticles was slightly affected (11% size variation using HA_4.8_-DPPE) by the freeze-drying process, thanks to the presence of HA-DPPE in the formulations. Comparable results were achieved for liposomes, which showed a size variation below 20% (Figure 6). All the resuspended formulations were stable during 4-weeks storage at 4 °C (see Figure S1 for PLGA 50:50 nanoparticles prepared with HA_4.8_-DPPE or HA_14.8_-DPPE).

Concerning zeta potential values, the addition of HA-DPPE conjugates lowered the surface charge of the nanocarriers (between −53 mV and −40 mV for PLGA nanoparticles and between −43 mV and −31 mV for liposomes) due to the presence of negatively charged functional groups of HA. After lyophilization, the overall obtained resuspended formulations maintained the strongly negative zeta potential, thus guaranteeing colloidal stability through electrostatic repulsion forces, according to the Derjaguin–Landau–Verwey–Overbeek (DLVO) theory [51].

**Figure 7:**
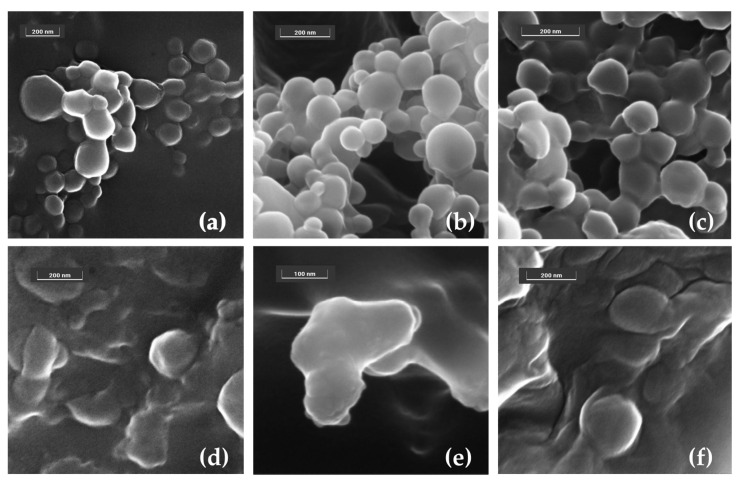
FESEM representative images of HA_4.8_-DPPE/PLGA 75:25 nanoparticles (**a**–**c**) and HA_4.8_-DPPE-liposomes (**d**–**f**) in their as prepared form (**a**,**d**), after freeze drying (**b**,**e**) and after resuspension (**c**,**f**). Images collected at 10 kV with the In-Beam SE detector. Instrumental magnification: 200,000× (**a**), 270,000× (**d**), 300,000× (**b**,**f**), 350,000× (**c**), and 550,000× (**e**), respectively.

FESEM analyses carried out on HA_4.8_-DPPE/PLGA 75:25 nanoparticles, and HA_4.8_-DPPE-liposomes further validated the beneficial effect of HA-DPPE in the formulations (Figure 7). In particular, HA_4.8_-DPPE-associated nanoparticles and liposomes in their as prepared form appear as embedded in a matrix formed upon drying of the solution. Moreover, HA_4.8_-DPPE/PLGA 75:25 nanoparticles showed an almost spherical shape (a), whereas HA_4.8_-DPPE-liposomes displayed a slightly globular shape (d) due to the drying step carried out to prepare the sample for the measurements (Figure 7). The samples were also examined after the freeze-drying process (b,e) as well as after further resuspension (c,f); the results showed that both morphology and size were maintained, which indicates that (i) HA-DPPE-associated lyophilized nanoparticles and liposomes can be redispersed after freeze drying, and (ii) the addition of HA-DPPE effectively enhanced the stability of the nanosystems.

The ability of HA conjugated on a liposome surface to act as a cryoprotectant has been previously reported by Peer et al. [52]: HA was linked to the surface of preformed liposomes by covalent conjugation. However, we used a different approach since the preformed HA-DDPE conjugates were added during liposome preparation, and the presence of the lipid anchor permitted the insertion of the conjugate into the bilayer [30,53]. To the best of our knowledge, for the first time, the insertion of these conjugates is described for polymer nanoparticles and proposed as cryoprotectants.

## 4. Conclusions

This comparative study confirms the crucial importance of the choice of a suitable cryoprotectant to obtain a redispersible lyophilized nanoparticle product. Indeed, the efficacy of the process strongly depends not only on the nature and concentration of the cryoprotective agent but also on the type of nanocarrier (lipidic or polymeric, with or without PEG or HA). As a consequence, the process conditions have to be selected by testing different experimental settings. Moreover, this type of study has to be repeated for each modification in the nanocarrier composition, e.g., the addition of an active principle or a fluorescent dye. Concerning our results, only the highest percentages (10–20%) of sucrose and trehalose showed to be able to act as cryoprotectant agents for both PLGA nanoparticles and liposomes.

Besides the classical saccharides used during the freeze-drying process, this work proposes the use of HA of different molecular weights as either a free molecule or conjugated to a phospholipid to achieve nanocarrier lyophilization. Although a moderate effect was observed for free HA-associated liposomes, more interesting are the results with HA-DPPE conjugates for both PLGA nanoparticles and liposomes: thanks to the lipophilic moiety, these molecules showed to be able to anchor to the PLGA matrix or to the liposome bilayer and to stably associate HA to the nanocarrier surface; in this way, HA-DPPE-decorated nanoparticles and liposomes can be resuspended after freeze-drying. This approach is particularly interesting since the HA moiety could act not only as a cryoprotectant agent but also as a targeting molecule towards the CD44 receptor, which is highly expressed in several cancer cells. Future steps will concern the evaluation of the ability of resuspended HA-associated nanoparticles and liposomes to target the CD44 receptor.

## Figures and Tables

**Table 1:** Mean hydrodynamic diameter and PDI of PLGA formulations before and after the freeze-drying process (*n* = 3).

		Before Freeze Drying		After Freeze Drying	
Formulation	Cryoprotectant % (*w*/*w*)	Hydrodynamic Diameter (nm)	PDI	Hydrodynamic Diameter (nm)	PDI
PLGA 75:25	Sucrose 10	136	0.086	140	0.120
PLGA 75:25	Sucrose 20	127	0.005	132	0.172
PLGA 75:25	Trehalose 10	144	0.072	143	0.090
PLGA 50:50	Glucose 10	131	0.177	141	0.111
PLGA 50:50	Sucrose 20	124	0.087	129	0.106
PLGA 75:25/PEG_2000_-PLGA	Glucose 20	104	0.090	147	0.338
PLGA 75:25/PEG_2000_-PLGA	Trehalose 5	85	0.080	132	0.351
PLGA 50:50/PEG_2000_-PLGA	Glucose 20	105	0.083	124	0.139

**Table 2:** Mean hydrodynamic diameter and PDI of liposomes before and after freeze-drying using a disaccharide:lipid ratio of 5:1 (*w*/*w*) (*n* = 3).

			Before Freeze Drying		After Freeze Drying	
Formulation	Cryoprotectant	Addition Method	Hydrodynamic Diameter (nm)	PDI	Hydrodynamic Diameter (nm)	PDI
Liposomes	Trehalose	Out *	204	0.163	218	0.190
Liposomes	Trehalose	In/Out **	210	0.139	221	0.143
Liposomes	Sucrose	Out *	200	0.138	190	0.163
Liposomes	Sucrose	In/Out **	201	0.165	204	0.208

* cryoprotectant added to the final liposomal suspension. ** cryoprotectant added during the lipid film hydration and in the final liposomal suspension.

## Data Availability

The data presented in this study are available from the corresponding author upon reasonable request.

## Supplementary Materials

The following supporting information can be downloaded at: https://www.mdpi.com/article/10.3390/ma16031212/s1.
