# Supplementary material for: Freeze Drying of Polymer Nanoparticles and Liposomes Exploiting Different Saccharide-Based Approaches
Source: Materials (Basel). 2023 Jan 31;16(3):1212. doi: 10.3390/ma16031212 (PMC9921637; doi:10.3390/ma16031212)
Supplement: Supplementary file 1 [file materials-16-01212-s001.zip › materials-2150845-supplementary.pdf]

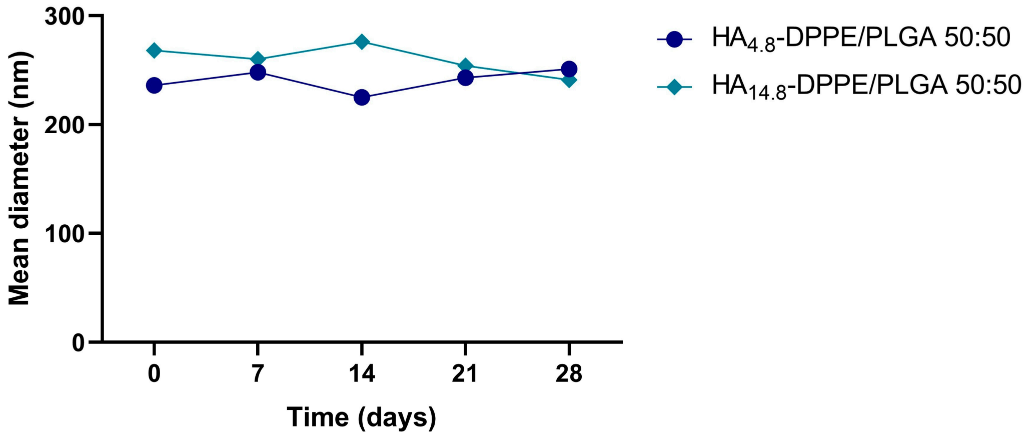

Figure S1. Mean hydrodynamic diameter of PLGA 50:50 nanoparticles prepared with $HA_{4.8}$-DPPE or $HA_{14.8}$-DPPE as a function of time (*n*=3, S.D.<10% for all samples).
